# Fabrication of Sandwiched NiCo-Layered Double Hydroxides/Carbon Nanoballs for Sustainable Energy Storage

**DOI:** 10.3390/polym16142005

**Published:** 2024-07-12

**Authors:** Thirukumaran Periyasamy, Shakila Parveen Asrafali, Seong-Cheol Kim, Jaewoong Lee

**Affiliations:** 1 Department of Fiber System Engineering, Yeungnam University, Gyeongsan 38541, Republic of Korea; thiru.kumaran999@gmail.com (T.P.); shakilaasraf@gmail.com (S.P.A.); 2 School of Chemical Engineering, Yeungnam University, Gyeongsan 38541, Republic of Korea; sckim07@ynu.ac.kr

**Keywords:** carbon nanoballs, bimetal oxides, layered double hydroxides, energy storage

## Abstract

This study presents a promising method for creating high-performance supercapacitor electrodes. The approach involves crafting a unique composite material—nickel-cobalt-layered double hydroxides (NiCo-LDH) grown on carbon nanoballs (CNBs). This is achieved by first creating a special carbon material rich in oxygen and nitrogen from a polybenzoxazine source. At first, eugenol, ethylene diamine and paraformaldehyde undergo Mannich condensation to form the benzoxazine monomer, which undergoes self-polymerization in the presence of heat to produce polybenzoxazine. This was then carbonized and activated to produce CNBs containing heteroatoms. Then, through a hydrothermal technique, NiCo-LDH nanocages are directly deposited onto the CNBs, eliminating the need for complicated templates. The amount of CNBs used plays a crucial role in performance. By optimizing the CNB content to 50%, a remarkable specific capacitance of 1220 F g^−1^ was achieved, along with excellent rate capability and impressive cycling stability, retaining 86% of its capacitance after 5000 cycles. Furthermore, this NiCo-LDH/CNB composite, when combined with active carbon in a supercapacitor configuration, delivered outstanding overall performance. The exceptional properties of this composite, combined with its simple and scalable synthesis process, position it as a strong contender for next-generation sustainable energy storage devices. The ease of fabrication also opens doors for its practical application in advancing energy storage technologies.

## 1. Introduction

In the quest for a sustainable future, we are moving away from dwindling fossil fuels and turning towards innovative energy storage solutions. Supercapacitors, boasting eco-friendliness, exceptional cycle life, and impressive power delivery, have emerged as strong contenders. However, their Achilles’ heel lies in their relatively low energy density [1,2,3]. To overcome this limitation and create high-performance supercapacitors, researchers are exploring asymmetric designs. These asymmetric supercapacitors leverage the best of both worlds: a battery-type electrode for the positive side, known for high energy density, and a carbon-based negative electrode, celebrated for its excellent power delivery. This strategic combination unlocks the potential for a significant leap in both power and energy density simultaneously. The key to achieving this lies in developing high-performance battery-type electrode materials. The better the performance of this positive electrode, the more impressive the overall properties of the asymmetric supercapacitor become. In essence, by unlocking the full potential of the battery-type electrode, we can push the boundaries of asymmetric supercapacitor performance, paving the way for a future powered by efficient and sustainable energy storage [4,5,6,7].

Transition metal hydroxides, particularly layered double hydroxides (LDHs), have emerged as promising materials for asymmetric supercapacitors due to their battery-like energy storage capabilities. LDHs boast a unique structure with positively charged brucite-like layers sandwiched between negatively charged interlayer anions, offering excellent anion-exchange stability and a well-defined layered architecture. This structure has garnered significant research interest not only in supercapacitors but also in diverse fields like catalysis, separation, and biotechnology. Furthermore, incorporating transition metals into LDHs creates hybrids with high redox activity, affordability, and extended cycle life, making them ideal candidates for battery-type electrodes. However, inherent limitations hinder LDH performance: low intrinsic conductivity and a tendency to aggregate. These drawbacks significantly hamper energy density and rate capability. Researchers are actively exploring strategies to overcome these limitations. They are focusing on the rational design and fabrication of LDH nanostructures and the creation of LDH-conductive material composites, paving the way for significant advancements in the electrochemical properties of these promising materials [8,9,10,11].

Supercapacitors (SCs) primarily rely on carbon-based electrode materials, resulting in solely electrostatic energy storage via electrical double-layer capacitance (EDLC). However, significant improvements in energy and power density can be achieved by combining EDLC with pseudocapacitance. Pseudocapacitive materials leverage reversible faradaic reactions at the electrode-electrolyte interface, boosting the specific capacitance. To this end, researchers have explored a diverse range of electrode materials for SCs, encompassing organic, inorganic, and polymeric substances. These include activated carbons, carbon nanotubes (CNTs), transition metal oxides/sulfides/hydroxides, and conducting polymers. The selection of the most suitable material hinges on critical factors like the specific surface area, pore size, the presence of a 3D hierarchical structure, the capability for additional redox reactions, and the electrical conductivity of the electrode material itself. By carefully considering these factors, scientists can design SC electrodes that offer optimal performance for various applications [12,13,14,15,16,17].

Hollow micro/nanostructures, captivating researchers for their unique properties, are essentially tiny, precisely crafted shells with a distinct empty interior. This design grants them several advantages: remarkably low density, a large surface area for interaction, and efficient pathways for materials to move within the structure. While scientists have been successful in creating these intricate structures using templates, the process can be quite complex. A recent breakthrough involves utilizing a specific metal-organic framework (ZIF-67) as a platform for generating diverse hollow architectures while maintaining their original shape. This approach holds promise for simpler fabrication. Building on this, researchers have employed a two-step diffusion method to create hollow prisms out of tiny cobalt sulfide subunits a promising development for lithium-ion battery technology. Further advancements include the creation of complex “box-in-box” structures with exceptional electrical properties, ideal for energy storage applications. However, a key hurdle remains—the inherently low conductivity of these materials limits their full potential. By addressing this conductivity issue through strategic design, scientists could unlock a new era of high-performance electrodes with tailored properties [18,19,20].

In the ongoing quest for better energy storage solutions, researchers are focusing on merging battery-type materials with highly conductive carbonaceous materials like carbon nanotubes, graphene, and active carbon. Graphene, in particular, has emerged as a superior platform for growing functional nanomaterials due to its exceptional matrix properties [21,22,23]. These graphene-based nanocomposites, combining the high specific capacitance of battery-type materials with graphene’s superior conductivity, have attracted significant interest. Graphene also helps prevent the restacking of these nanomaterials, maximizing the utilization of their active sites. However, incorporating hollow nanostructures, a key feature for optimizing nanoparticles, onto graphene sheets remains a challenge. The novelty of the work lies in creating both electrical double layer capacitance (EDLC) and pseudocapacitance (PC). As polybenzoxazine acts as a source for producing hetero-doped carbon materials, EDLC and a minimum amount of PC are expected from these carbon materials. The inclusion of bimetallic materials helps to increase the pseudocapacitance, thereby increasing the overall energy storage of the NiCo-LDH/CNB composites. This study presents a novel method for synthesizing and characterizing NiCo-LDH hollow nanocages deposited on carbon nanoballs, utilizing a high-temperature, structure-induced, anisotropic chemical etching process. The synthesis involves first obtaining a metal-organic framework/CNB composite through a precipitation reaction, followed by an acidic etching step that transforms the template into a hollow structure. The resulting NiCo-LDH/CNB composite demonstrates exceptional performance as a battery-type electrode due to its unique “sandwich-like” hollow architecture. This design offers several advantages: first, the interconnected network of LDH nanosheets within the hollow cage provides ample active sites for electrolyte interaction. Second, the hollow structure effectively accommodates the volume changes that occur during charge and discharge cycles. Finally, the CNB core serves a dual purpose: it acts as a base for anchoring the hollow LDH nanocages while also providing efficient pathways for electron transport. This unique architecture facilitates the uniform growth of the hollow nanocages on the CNB, which are then encapsulated within the cages. This configuration enables both rapid electron transfer by minimizing the distance between the ions need to travel within the electrode. This study further explores the impact of CNB content on the electrochemical performance, comparing it to standalone NiCo-LDH nanocages, highlighting the crucial role of CNB. By combining these advantages, the NiCo-LDH nanocages/CNB composite presents itself as a promising and practical candidate for high-performance supercapacitors.

## 2. Materials

Eugenol and paraformaldehyde were obtained from Sigma Aldrich, Seoul, Republic of Korea, while, ethylene diamine, potassium hydroxide, sodium hydroxide, nickel nitrate hexahydrate, cobalt nitrate tetrahydrate, polyvinylidene fluoride (PVDF), N, N-dimethylformamide (DMF) and ethanol were supplied from Duksan Chemicals Co., Ltd., Ansansi, Republic of Korea. All the chemicals were used without further purification.

## 3. Methods

### 3.1. Synthesis of Polybenzoxazine Based Carbon Nano Balls

**Scheme 1:**
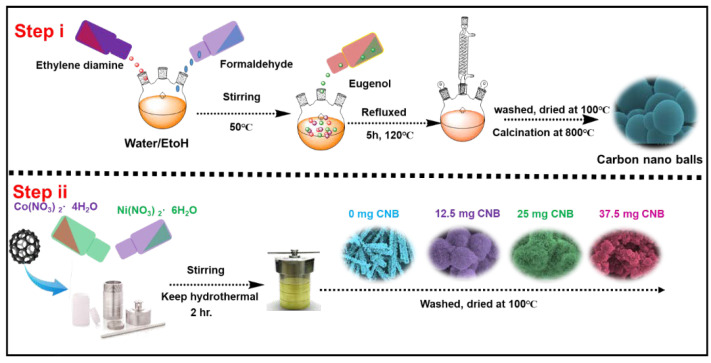
Schematic representation showing the formation of CNB and NiCo-LDH/CNB.

Eugenol and ethylene diamine based benzoxazine monomer, Eu-Bzo, was synthesized for the production of activated carbon materials. Eugenol, ethylenediamine, and paraformaldehyde underwent a Mannich condensation reaction in a 2:1:4 molar ratio using a 1:1 water–ethanol mixture at 120 °C for 3 h. The formed precipitate after the completion of the reaction was isolated through precipitation and drying to obtain the precursor, Eu-Bzo. This Eu-Bzo monomer was then subjected to a stepwise curing process, gradually increasing the temperature to a maximum of 250 °C. This curing process transformed the monomer into a self-curing thermoset polymer, denoted as poly (Eu-Bzo). Finally, the poly (Eu-Bzo) was carbonized under a nitrogen atmosphere at 600 °C, followed by activation with KOH in a furnace at 600 °C, creating the final material with unique properties. This process yielded activated carbon materials containing heteroatoms, denoted as CNB (Scheme 1).

### 3.2. Synthesis of Ni-Co LDH/CNB Composites

Three different NiCo-LDH/CNB composites were produced through hydrothermal process by varying the amount of NiCo-LDH and CNB. The process begins by mixing the CNB in ethanol with nickel and cobalt nitrate salts, and kept for hydrothermal reaction at 120 °C for 2 h, which transforms the powder from purple to light green. After filtration and drying, the final composites were obtained. For ease of understanding the composites were denoted as NiCo-LDH/25 CNB, NiCo-LDH/50 CNB and NiCo-LDH/75 CNB. To see the effect of CNB addition, NiCo-LDH without CNB was prepared and denoted as NiCo-LDH/0 CNB (Scheme 1).

## 4. Instrumentation

X-ray diffraction (XRD): A PANalytical X’Pert3 MRD diffractometer was employed to identify the crystalline phases present in the sample. The instrument utilized monochromatized Cu Kα radiation with a wavelength (λ) of 1.54 Å. The analysis was conducted under a voltage of 40 kV and a current of 30 mA. The data were collected over a 2θ range of 10°–90°.

Field-emission scanning electron microscopy (FESEM) with Energy-Dispersive X-ray Spectroscopy (EDS): A Hitachi S-4800 instrument (Ibaraki, Japan) operating at an accelerating voltage of 4 kV was used for a combined analysis. FESEM provided high-resolution images revealing the morphology (shape and size) of the material, while EDS enabled the determination of its elemental composition.

High-Resolution Transmission Electron Microscopy (HRTEM): Detailed structural information at the atomic level was obtained using an FEI-Tecnai TF-20 microscope operating at 120 kV. HRTEM provided high-resolution images that allowed for the visualization of the material’s crystal structure.

X-ray Photoelectron Spectroscopy (XPS): A K-Alpha spectrometer from Thermo Scientific (Waltham, MA, USA) was utilized to investigate the elemental composition and chemical state of the sample’s surface. The high-resolution XPS spectra were further analyzed using CasaXPS software, version number-2.3.26 for deconvolution.

## 5. Electrochemical Measurements

The electrochemical properties of Ni-Co LDH/CNB material using a three-electrode system with a CorrTest-CS350 workstation in 1 M KOH electrolyte. Cyclic voltammetry (CV) explored the material’s behavior within a potential range of 0.0–0.8 V at various scan rates. Galvanostatic charge–discharge (GCD) assessed its charge storage capacity at different current densities between 0.0 and 0.5 V. Electrochemical impedance spectroscopy (EIS) examined the electrode kinetics and resistance in the frequency range of 0.01 kHz–100 kHz. All measurements were conducted at room temperature. A specific capacitance equation was then used to analyze the GCD data. the following equation:

Cs = IΔt/mΔV(1)

The equation relates the specific capacitance (Cs) of an electroactive material to the current (I), discharge time (Δt), potential window (ΔV), and mass (m) of the material during a charge–discharge process.

## 6. Crystallographic Structure Analysis of the Composites

**Figure 1:**
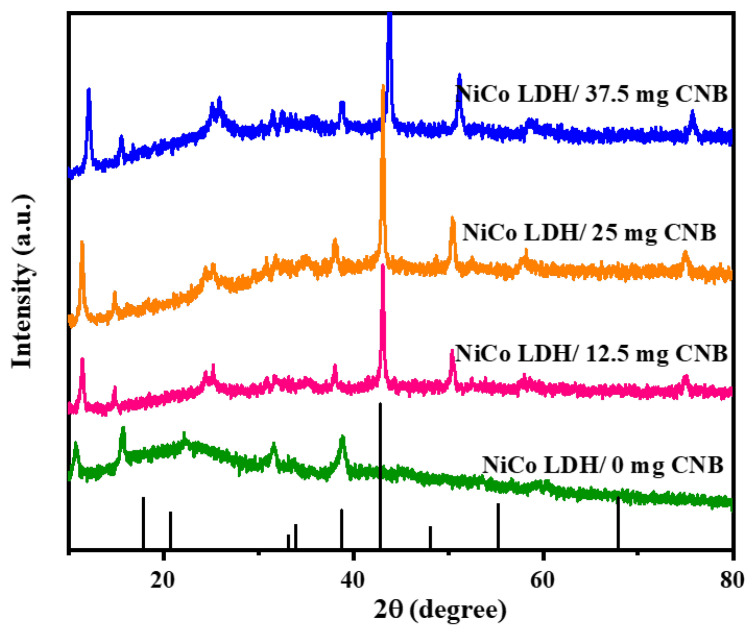
XRD spectrum of NiCo-LDH/CNB composites.

X-ray diffraction (XRD) measurements were employed to investigate the crystallographic structure of Ni-Co-layered double hydroxide (LDH) composites intercalated with varying amounts of carbon nanoballs. The obtained XRD patterns (Figure 1) displayed excellent agreement with simulated peaks reported in the literature, signifying the high purity of the synthesized NiCo-LDH/CNB composites. All observed diffraction peaks could be assigned to the NiCo-LDH phase (JCPDS no. 40-0416), confirming its successful formation and the absence of any impurities. A sharp peak at 26° indicated the presence of highly graphitized CNB, while a broader peak at the same position suggested its exfoliated nature [24,25,26].

Further analysis revealed consistent diffraction peaks for the NiCo-LDH phase across all samples, irrespective of the CNB content. This confirmed the successful integration of the LDH structure within the composites. Peaks corresponding to CNB emerged for all the composites loaded with CNB. This suggests the uniform deposition of hierarchical NiCo-LDH nanocages on the CNB surface, effectively hindering CNB restacking. However, increased CNB content resulted in agglomeration, as evidenced by the intensified CNB-related XRD peaks. Interestingly, the NiCo-LDH/CNB composites exhibited broadened and weakened diffraction peaks. This can be attributed to the formation of numerous, well-dispersed, nanoscale LDH crystallites within the composites. This nanoscale structure implies a high surface area and potentially improved material performance due to the fine distribution of the LDH phases throughout the composite. These characteristics hold promise for various applications.

## 7. Microscopic Examination of the Materials

**Figure 2:**
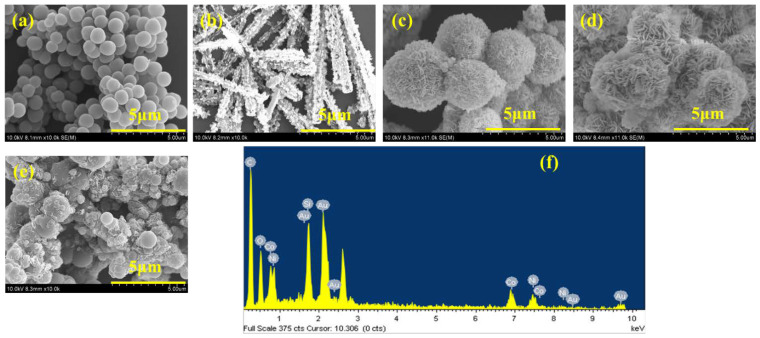
SEM images of (**a**) CNB; (**b**) NiCo-LDH; (**c**) NiCo-LDH/25 CNB; (**d**) NiCo-LDH/50 CNB; (**e**) NiCo-LDH/75 CNB; and (**f**) EDX spectrum of NiCo-LDH/50 CNB.

Scanning electron microscopy (SEM) was employed to investigate the morphology of the synthesized materials, including pristine CNB, NiCo-LDH and NiCo-LDH/CNB composites. The high-resolution SEM images (Figure 2) revealed distinct characteristics for each material. Pristine CNB exhibited a unique structure composed of uniformly dispersed, ball-like nanoparticles with a smooth texture. Interestingly, these nanoparticles assembled to form a porous network, creating a micro-porous surface. The average diameter of these CNB particles ranged from 100 to 500 nm. In contrast to the spherical CNB, NiCo-LDH displayed a star-shaped rod-like morphology with a well-defined thickness of approximately 30 nm. These rods are vertically oriented and interconnected, forming a three-dimensional network. The SEM analysis provided valuable insights into the morphological differences between CNB, NiCo-LDH, and their composites. Notably, the NiCo-LDH content significantly influenced the composite structure.

The morphology of the composites played a crucial role in their potential applications. NiCo-LDH/25 CNB displayed a higher concentration of flake-like NiCo-LDH structures deposited on the CNB base. However, this high loading resulted in some degree of agglomeration, likely hindering the material’s performance. Conversely, NiCo-LDH/50 CNB exhibited a more balanced distribution of flake-like NiCo-LDH structures across the CNB surface. This uniform distribution was identified as an ideal configuration for maintaining the structural integrity and maximizing the functional properties of the composite. In NiCo-LDH/75 CNB, the lower NiCo-LDH content resulted in sparse deposition of flake-like structures. This composite suffered from both agglomeration of CNB particles due to the excessive CNB content and insufficient coverage of CNB by NiCo-LDH. The findings suggest that the NiCo-LDH/50 CNB composite achieved an optimal morphology, offering a well-balanced combination of CNB and NiCo-LDH for superior electrochemical performance.

**Figure 3:**
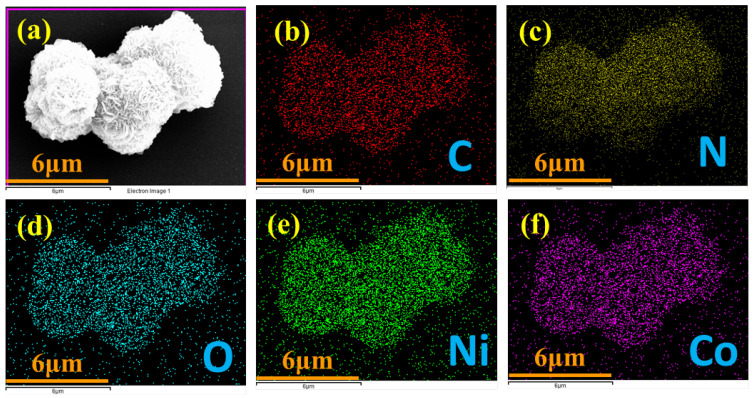
Elemental mapping of (**a**) NiCo-LDH/50 CNB; and (**b**–**f**) individual elements.

An in-depth analysis using elemental mapping techniques (illustrated in Figure 3a–f) confirmed the successful integration of various elements within the NiCo-LDH/CNB composite. Carbon (C) and nitrogen (N) signatures originated from the porous carbon substrate, while cobalt (Co) and nickel (Ni) stemmed from the bimetallic inclusions. Oxygen (O) was attributed to both the porous carbon (CNB) and the bimetallic oxides. Importantly, these elements were uniformly dispersed throughout the composite, as shown by the individual mapping images. This homogenous distribution not only guarantees the structural integrity and consistent material properties but also signifies the preservation of the spherical core structure during synthesis, indicative of remarkable material stability. This even distribution of elements is paramount for the composite’s performance, as it implies efficient interaction and synergy between its various components—a key factor for potential applications in catalysis and electronics [27,28,29].

**Figure 4:**
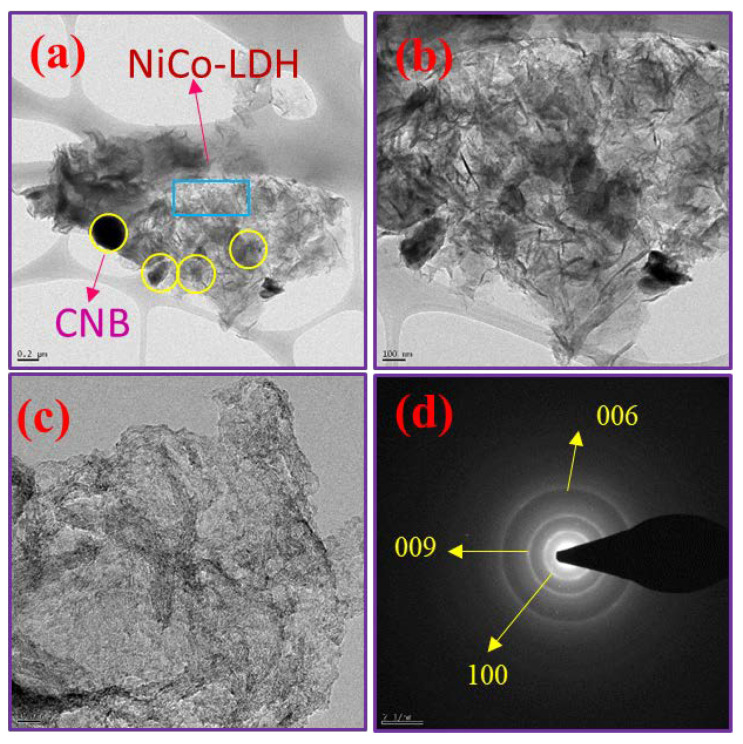
(**a**–**c**) HR-TEM images and (**d**) SAED pattern of NiCo-LDH/50 CNB.

The NiCo-LDH/CNB composite’s detailed morphological characteristics were analyzed using Transmission Electron Microscopy (TEM). This analysis revealed the presence of hierarchical nanocages on the CNB, as shown in Figure 4a–d. These nanocages are easily distinguishable as they are attached to the CNB, featuring interlinked petals at the base and the nanocages themselves at the top. This structural formation likely results from the nucleation process, where ions nucleate on the CNB surface and subsequently grow into spherical shapes over time. The ion-exchange reaction that follows leads to the development of small petals and nanocages. This hierarchical structure is particularly advantageous as it provides an extensive active surface area, enhancing the material’s ability to interact with the electrolyte and store significant amounts of electrical energy, making it highly suitable for energy storage applications. Further meticulous examination of the nanocages’ edges reveals that their shells are composed of interconnected nanosheets with ultrathin thicknesses. Despite these intricate details, the primary shape of the NiCo-LDH remains largely unchanged, aligning with the observations from scanning electron microscopy (SEM). Additionally, the Selected Area Electron Diffraction (SAED) pattern in Figure 4d confirms the presence of distinct phases of bimetallic oxides, further validating the composite’s structural integrity and composition.

## 8. X-ray Photoelectron Spectroscopy Analysis of the Composite

**Figure 5:**
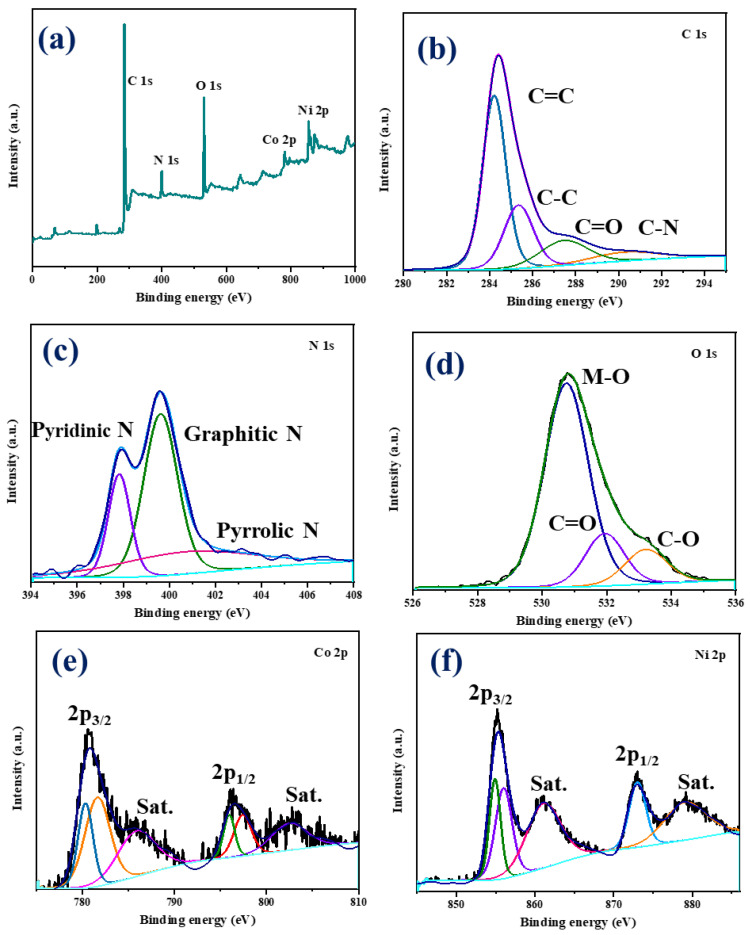
XPS spectra of NiCo-LDH/50 CNB showing (**a**) survey spectrum; and (**b**–**f**) deconvoluted spectrum.

**Scheme 2:**
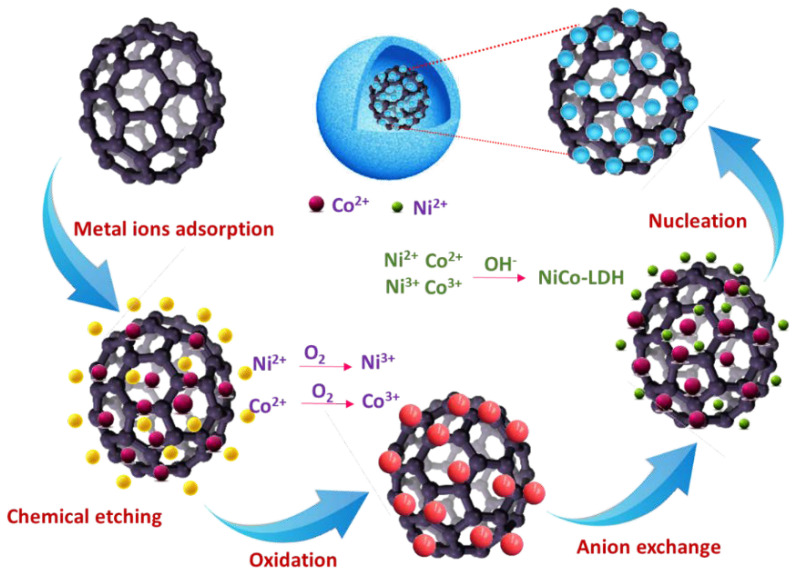
Schematic illustration of the possible mechanism reaction involved in forming NiCo-LDH.

To understand the elemental composition of the NiCo-LDH/CNB composite material, a technique called X-ray Photoelectron Spectroscopy (XPS) was employed. This analysis confirmed the presence of several key elements: carbon (C), nitrogen (N), oxygen (O), cobalt (Co), and nickel (Ni) (Figure 5a). To gain a deeper understanding of the chemical environment surrounding each element, high-resolution XPS spectra were obtained (Figure 5b–f). The C1s spectrum (Figure 5b) is a composite of four distinct peaks. The peak at 284.1 eV signifies the presence of carbon atoms engaged in aromatic ring structures, specifically C=C and C-C bonds. A higher binding energy peak at 287.1 eV corresponds to carbonyl carbon (C=O/C-O), while another peak at 290.5 eV represents C-N bonds. Interestingly, the intensity of the carbonyl carbon peak is greater compared to the C-N peak, suggesting that the carbonization process might have led to the formation of additional C-O bonds on the carbon surface [30,31,32]. The N1s spectrum (Figure 5c) consists of three distinct peaks centered at 397.8 eV, 399.6 eV, and 401.3 eV. These peaks are attributed to three different types of nitrogen species within the material: pyridinic, graphitic, and pyrrolic nitrogen, respectively. This distribution of peaks confirms the successful incorporation of nitrogen both within the carbon structure and on its surface [33,34,35]. The O1s spectrum (Figure 5d) can be deconvoluted into three separate peaks. The most prominent peak at 530.8 eV originates from metal–oxygen–metal (M-O-M) bonds, a signature of the spinel lattice structure. Two lower-intensity peaks are observed at 530.0 eV and 533.2 eV. These are attributed to surface-adsorbed carbonyl oxygen, chemisorbed oxygen, and coordinated lattice oxygen, respectively. It is noteworthy that the XPS analysis reveals a higher proportion of M-O bonds within the spinel structure compared to the carbonyl bonds associated with the carbon framework. The Co 2p spectrum (Figure 5e) clearly exhibits two sets of peaks corresponding to Co^2+^ and Co^3+^ ions. The Co^2+^ species are identified by peaks at 781.7 eV (2p₃/₂) and 797.4 eV (2p₁/₂), while Co^3+^ displays peaks at 780.3 eV (2p₃/₂) and 796.0 eV (2p₁/₂). The presence of satellite peaks further confirms the coexistence of both oxidation states. Similarly, the deconvoluted Ni 2p spectrum (Figure 2f) reveals two distinct sets of peaks attributable to Ni^2+^ and Ni^3+^. The binding energies for Ni^2+^ are 854.9 eV (2p₃/₂) and 872.9 eV (2p₁/₂), while those for Ni^3+^ are 856.0 eV (2p₃/₂) and 873.1 eV (2p₁/₂). Additionally, shake-up satellite peaks are observed for Ni^3+^. XPS analysis confirms the presence of carbon, nitrogen, oxygen, cobalt, and nickel in the NiCo-LDH/CNB composite [36,37,38,39]. The C1s spectrum suggests the formation of additional C-O bonds during the carbonization process. The N1s spectrum confirms the successful incorporation of nitrogen within the carbon structure and on its surface. The O1s spectrum indicates a dominance of M-O bonds within the spinel structure compared to the carbonyl bonds in the carbon framework. These findings demonstrate the presence of both Co and Ni in multiple oxidation states within the NiCo-LDH/CNB composite. This unique combination, along with the oxygen and nitrogen functional groups incorporated into the heteroatom-doped carbon framework, creates a favorable environment for rapid electron transfer. This efficient electron transport is believed to significantly enhance the electrical conductivity of the overall material (Scheme 2).

## 9. Electrochemical Studies of the Composites

**Figure 6:**
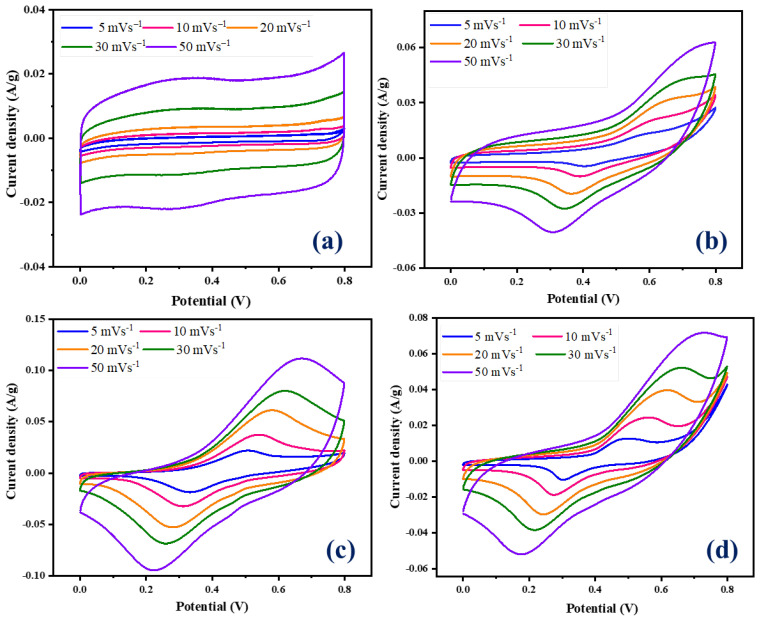
CV graphs of (**a**) CNB; (**b**) NiCo-LDH/CNB (25%); (**c**) NiCo-LDH/CNB (50%); and (**d**) NiCo-LDH/CNB (75%).

The prepared NiCo-LDH/CNB composites were used as working electrodes, and their charge storage phenomenon was evaluated using electrochemical measurements. We employed cyclic voltammetry (CV), galvanostatic charge–discharge (GCD), and electrochemical impedance spectroscopy (EIS) in a three-electrode configuration with 1 M KOH electrolyte. A mercury/mercury oxide (Hg/HgO) reference electrode, a platinum (Pt) counter electrode, and the working electrode fabricated using NiCo-LDH/CNB composites completed the setup. Figure 6a showcases the CV curve of pure CNB, revealing a box-like shape at high scan rates. This is characteristic of electrical double-layer capacitance (EDLC), where charge is stored on the material’s surface. Notably, there are no prominent redox peaks at lower scan rates, which is typical for carbon-based materials. Conversely, the CV curves for NiCo-LDH/CNB composites (Figure 6b–d) display distinct redox peaks at various scan rates, including an oxidation peak around 0.4 V and a reduction peak near 0.2 V. The presence of these persistent redox peaks, even at a high scan rate of 50 mV s^−1^, signifies the pseudocapacitive behavior of the NiCo-LDH/CNB electrodes. This suggests that incorporating NiCo-LDH into the CNB matrix enhances the overall capacitance. The combination offers not only EDLC from CNB but also pseudocapacitance from the bimetallic oxide (NiCo-LDH). This synergistic effect between the two materials leads to improved charge storage capabilities of the composite electrodes [40,41,42,43,44,45,46].

**Figure 7:**
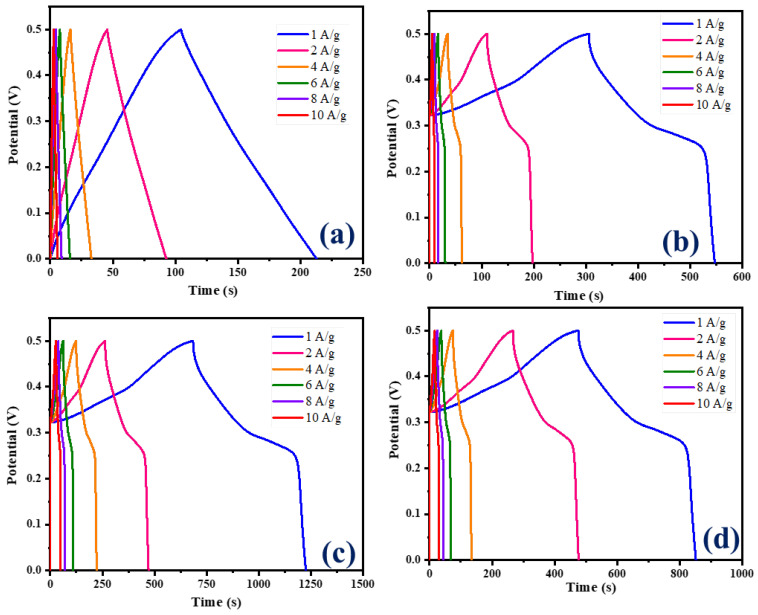
GCD graphs of (**a**) CNB; (**b**) NiCo-LDH/CNB (25%); (**c**) NiCo-LDH/CNB (50%); and (**d**) NiCo-LDH/CNB (75%).

The galvanostatic charge–discharge (GCD) curves for CNB and Ni-Co LDH/CNB composites at varying current densities are depicted in Figure 7a–d. These figures illustrate the performance of the materials under different conditions. The GCD curves of CNB displayed an ideal triangular shape, due to EDLC, whereas the GCD curve of all the NiCo-LDH/CNB composites displayed a distorted triangular shape, a behavior attributed to pseudocapacitive effects. Moreover, the Ni-Co LDH/CNB composite exhibits an asymmetric triangular shape in its GCD curves, which contrasts with the more symmetric triangular shapes observed in the GCD curves of the CNB. This difference in shape highlights the distinct electrochemical characteristics and charge-storage mechanisms of the two materials [47,48,49].

**Figure 8:**
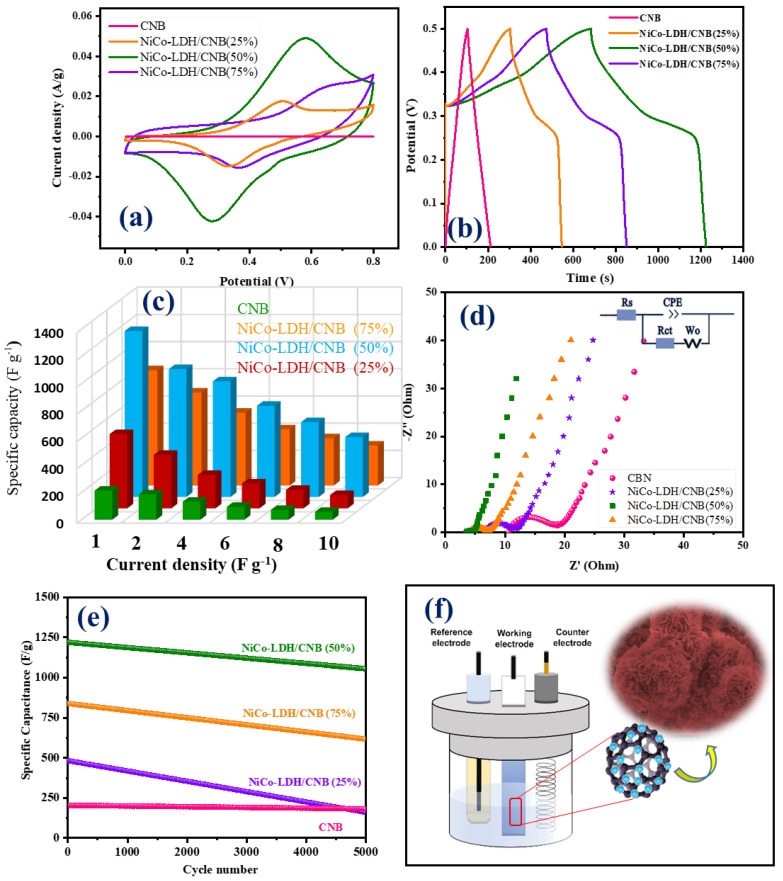
(**a**) CV graphs; (**b**) GCD graphs; (**c**) Specific capacity; (**d**) EIS spectra; and (**e**) cyclic stability of CNB and NiCo-LDH/CNB composites; (**f**) schematic of the electrochemical reaction.

The graphical representation in Figure 8a provides a comparative analysis of cyclic voltammetry (CV) curves between two distinct materials: carbon nanoballs and Ni-Co LDH/CNB composite. These curves were recorded under identical conditions, employing a scan rate of 20 mV s^−1^. On initial observation, each curve corresponding to NiCo-LDH/CNB composite exhibits discernible redox peaks, indicating the pseudocapacitive nature. However, a closer inspection reveals nuanced differences in the profiles, notably in the positioning of these redox peaks at different voltages. This variance can be attributed to variances in polarization behavior and the ohmic resistance experienced by the electrodes during the CV testing phase. Delving deeper, significant potential ranges between the redox peaks are discernible, particularly in CV curves featuring lower CNB mass percentages (25% and 50%). This phenomenon likely stems from the elevated resistance encountered in materials with reduced CNB content. Conversely, as the proportion of CNB exceeds 50%, a notable shift in redox peaks, coupled with a narrower potential range, becomes evident in the CV curves. This shift underscores a synergistic interplay between Ni-Co LDH and CNB, resulting in enhanced electrochemical performance.

The Ni-Co LDH/50 CNB composite notably exhibits the most extensive CV integrated area among all compositions, indicating a high specific capacitance at this optimal CNB mass. However, a subsequent increase in CNB mass leads to a decline in capacitance, presumably due to reduced Ni-Co LDH loading content, thereby limiting the faradic reaction sites available for interaction with the electrolyte. Nevertheless, even at 75% CNB content, the Ni-Co LDH composite displays pronounced redox peaks within a narrow potential range, indicative of superior conductivity and excellent reversibility. This enhanced electrochemical performance is corroborated by galvanostatic charge–discharge (GCD) curves, as depicted in Figure 8b. GCD curves are employed to assess the properties of all composites under a constant current density of 1 A g^−1^. It is notable that the potential range applied in charge–discharge curves is smaller than that in CV curves, possibly due to the oxidation evolution reaction. Notably, the discharge time of the Ni-Co LDH/50% CNB composite significantly surpasses that of pure CNB, affirming the augmented specific capacitance conferred by the Ni-Co LDH/CNB composite, consistent with the CV findings. This superiority can be attributed to the reduced resistance encountered by the electrode. As the CNB content increases, a dramatic reduction in internal resistance (IR drop) is observed, indicating the effective reduction in composite resistance and an improvement in charge transport and electron collection rates facilitated by CNB. Particularly, the internal resistance recorded for 75% of CNB is markedly lower compared to other compositions, likely attributed to the surplus CNB content reducing composite resistance. All GCD curves exhibit a characteristic plateau consistent with CV curves, indicative of pseudocapacitance. By calculating the discharge time, specific capacitances are determined to be 214 F g^−1^ for CNB, 546 F g^−1^ for NiCo-LDH/25 CNB, 1220 F g^−1^ for NiCo-LDH/50 CNB and 848 F g^−1^ for NiCo-LDH/75 CNB further affirming the pronounced performance of the Ni-Co LDH/CNB composite. To substantiate this hypothesis, Electrochemical Impedance Spectroscopy (EIS) measurements are conducted across a frequency range from 10 kHz to 0.01 Hz (Figure 8d). Remarkably, both the equivalent series resistance and charge transfer resistance of Ni-Co LDH/CNB are notably lower compared to pure CNB, underscoring the improved electrochemical properties of the former [50,51,52].

For comparative analysis, the electrochemical performances of CNB and other NiCo-LDH/CNB composites were examined. Notably, NiCo-LDH/50% CNB exhibits the highest specific capacitance with the lowest resistance among the four materials, underscoring the synergistic effect between CNB and NiCo-LDH in enhancing material performance. This highlights the importance of in situ growth for fabricating Ni-Co LDH/CNB composites, as opposed to synthesizing them through a mixture of NiCo-LDH and CNB, which yields comparatively unsatisfactory results. Moreover, the electrodes exhibit excellent durability, maintaining capacitances of 220 F g^−1^ for CNB and NiCo-LDH/25 CNB, 1115 F g^−1^ for NiCo-LDH/50 CNB, and 650 F g^−1^ for Ni-Co-LDH/75 CNB even after 5000 cycles. Cyclic stability measurements (Figure 8e) reveal percentage retention rates of 44%, 91%, and 76%, respectively, after 5000 cycles, further attesting to the robustness and longevity of the electrodes. Comparison of Electrochemical Performances for Various Supercapacitors in Table 1.

## 10. Conclusions

In summary, a novel method has been developed for creating hierarchical NiCo-LDH nanocages embedded with carbon nanoballs (CNB), aimed at enhancing the performance of energy storage devices. This innovative approach employs a straightforward in situ precipitation process followed by an ion-exchange reaction, which is highly conducive to large-scale production due to its simplicity and efficiency. The resulting composite material features a distinctive nanocage architecture, characterized by interconnected LDH nanopetals that are uniformly deposited on the CNB. This hierarchical structure, in combination with the excellent conductivity of the CNB, markedly improves ion mobility within the material. Consequently, this leads to accelerated charging times and superior capacitance, making the composite highly effective for use in battery-type electrodes. Experimental evaluations reveal that the composite demonstrates optimal electrochemical performance when the CNB content is maintained at 50%. Supercapacitors constructed with this NiCo-LDH/CNB composite, paired with activated carbon, exhibit outstanding properties such as high specific capacitance of 1220 F g^−1^ and remarkable long-term stability maintaining 91% retention rate after 5000 cycles for NiCo-LDH/50 CNB. These promising results underscore the potential of NiCo-LDH/CNB composites as a leading candidate for next-generation sustainable energy devices, particularly in applications requiring high-efficiency battery-type electrodes. The combination of facile synthesis, scalability, and enhanced electrochemical characteristics positions this composite as a significant advancement in the field of energy storage technology.

## Figures and Tables

**Table 1:** Comparison of Electrochemical Performances for Various Supercapacitors.

Positive Materials// Negative Materials	Specifi Capacitor F/g	Cycle No	Retention %	Refs.
Ni-Co DH//AC	1120	3000	80.7	[53]
MnCo-LDH@ Ni(OH)_2_//AC	2320	5000	90.9	[54]
NiCo_2_O_4_@ NiO//AC	1674	3000	89.0	[55]
CoNiFe-LDH/CNFs-0.5//AC	1203	2000	82.7	[56]
Ni-Co LDH from ZIF-67//AC	1530	1000	89.3	[49]
HCNs@NiCo@LDH/graphene	1749	3000	93.5	[57]
CBC-N2@LDH@0.4//CBC-N2	1943	2500	89.3	[57]
Ni-Co LDH/CNB	1220	5000	91.0	This work

## Data Availability

The data presented in this study are available in this article.
